# Rapid climate fluctuations over the past millennium: evidence from a lacustrine record of Basomtso Lake, southeastern Tibetan Plateau

**DOI:** 10.1038/srep24806

**Published:** 2016-04-19

**Authors:** Kai Li, Xingqi Liu, Ulrike Herzschuh, Yongbo Wang

**Affiliations:** 1 State Key Laboratory of Lake Science and Environment, Nanjing Institute of Geography and Limnology, Chinese Academy of Sciences, Nanjing, 210008, China; 2 University of Chinese Academy of Sciences, Beijing, 100049, China; 3 Beijing Key Laboratory of Resource Environment and GIS, College of Resource Environment and Tourism, Capital Normal University, Beijing, 100048, China; 4 Alfred Wegener Institute Helmholtz Centre for Polar and Marine Research, Research Unit Potsdam, Potsdam, 14473, Germany; 5 Institute of Earth and Environmental Science, University of Potsdam, Potsdam, 14476, Germany

## Abstract

Abrupt climate changes and fluctuations over short time scales are superimposed on long-term climate changes. Understanding rapid climate fluctuations at the decadal time scale over the past millennium will enhance our understanding of patterns of climate variability and aid in forecasting climate changes in the future. In this study, climate changes on the southeastern Tibetan Plateau over the past millennium were determined from a 4.82-m-long sediment core from Basomtso Lake. At the centennial time scale, the Medieval Climate Anomaly (MCA), Little Ice Age (LIA) and Current Warm Period (CWP) are distinct in the Basomtso region. Rapid climate fluctuations inferred from five episodes with higher sediment input and likely warmer conditions, as well as seven episodes with lower sediment input and likely colder conditions, were well preserved in our record. These episodes with higher and lower sediment input are characterized by abrupt climate changes and short time durations. Spectral analysis indicates that the climate variations at the centennial scale on the southeastern Tibetan Plateau are influenced by solar activity during the past millennium.

A fundamental aspect of climate is instability, which is one of the notable features of climate record in over geological periods. Long-term climate changes are always superimposed by abrupt changes^1^,^2^ and climate fluctuations^3^,^4^,^5^. The past millennium, covering the Medieval Climate Anomaly (MCA), Little Ice Age (LIA) and Current Warm Period (CWP), is a critical time frame that includes instrumental records as well as proxy records of past climate changes^6^. Research on the climate of the last two thousand years has produced numerous climate reconstructions from the continental to global scales^7^,^8^,^9^,^10^,^11^. However, the regional climate evolution at the sub-decadal to decadal time scales and the related forcing remain poorly understood.

The Tibetan Plateau (TP), the largest elevated landmass and the Third Pole of the Earth, is highly sensitive to global climate change^12^,^13^,^14^. The TP is a pilot region of climate fluctuations at time scales shorter than 1000 years^15^, with warm and cold stages that are 10–60 years ahead of regions elsewhere in China^11^,^15^. High resolution records have suggested that climate in the TP was variable during the past millennium^5^,^9^,^16^ and that rapid climate fluctuations at decadal to annual time scales can be recognized through well-dated and sensitive proxy records^5^,^16^. However, the characteristics and durations of these climate fluctuations have not yet been fully understood and require further study.

Basomtso Lake (93°53′42″–94°1′48″ E, 30°0′0″–30°2′55″ N, 3476 m a.s.l.) is located on the southeastern TP, which is mainly influenced by the Indian Summer Monsoon (ISM) (Fig. 1). Basomtso Lake is a freshwater lake with a pH of 7.2 and a salinity of 0.12 g L^−1^. The maximum depth occurrs in the western and eastern lake areas, with a depth of 120 m. The lake covers an area of 26 km^2^ and has a catchment area of 1209 km^2 ^17. The lake is surrounded by mountains with elevations of 4500–5200 m and slope angles of 45–55°. The vegetation is characterized by coniferous forests, shrubs and patches of meadows. The closest meteorological station is in Nyingchi, which is at an elevation of 3000 m a.s.l. and located 65 km southeast of Basomtso Lake. The meteorological station records for 1960–1964 AD and 1973–2012 AD indicate a mean July temperature (*T*_July_) of 15.9 °C, a mean January temperature (*T*_Jan_) of 0.9 °C and a mean annual precipitation (*P*_ann_) of 1130 mm (90% falling between April and October). The current Jiao-Guoguo and Zhong-Co glaciers are distributed in the Basomtso Lake basin (Fig. 1). Basomtso Lake is mainly fed by glacier melt water via fluvial runoff from the Basom and Nize Rivers and discharges into Yarlung-Tsangpo River through the Ba River and then Niyang River^17^.

## Results

### Chronology

The accelerator mass spectrometry (AMS) ^14^C dating results for the core sediment from Basomtso Lake (BSCW-1) are presented in Table 1. The six radiocarbon ages show a general linear correlation, confirming a continuous sedimentation process (Fig. 2a). The calibration and age-depth model were constructed with Bacon 2.2^18^ in R 3.2 software using Bayesian statistics to reconstruct Bayesian accumulation histories for the deposits (Fig. 2b). The model was performed using the default settings for lake sediments with 1-cm resolution. According to the age-depth model, the sedimentary rates of BSCW-1 ranged from 0.38 to 0.67 cm yr^−1^ with an average of 0.51 cm yr^−1^. Compared with other well-dated lacustrine records for the TP^19^,^20^,^21^, the sedimentary rates of Basomtso Lake during the past millennium were much higher. The higher sedimentary rate in Basomtso Lake is related to the high sediment yield from glacier activity as well as the high fluvial runoff fed by glacier melt water and precipitation.

### Variations in organic matter, grain size and magnetic susceptibility

The loss on ignition (LOI) of BSCW-1 ranges from 1.0% to 5.5% with an average of 2.6% (Fig. 3). The LOI values were relatively high for sediments that accumulated within the period of the 1080s–1140s and were low for the period of the 1140s–1800s. The LOI values were relatively high for the sediments deposited after 1800s, with an average of 3.5%. The variations in total organic carbon (TOC) and total nitrogen (TN) generally paralleled that of the LOI; i.e., higher TOC and TN values correspond to higher LOI values (Fig. 3). However, some periods characterized by lower LOI values (labelled 1–7) and brief high LOI values (labelled I–V), as well as rapid increases in the LOI, were recorded between the 1140s and the 1800s (Fig. 3).

The sediments in BSCW-1 are mainly composed of fine silt (4–32 μm) and clay (<4 μm), with average percentages of 63.8% and 28.1%, respectively (Fig. 3). The fine silt and clay fractions were high in the sediments that accumulated within the period from the 1080s to 1790s, which corresponds with a lower composition of coarse silt (32–63 μm) and sand (>63 μm). However, a brief increase in the fine silt, coarse silt and sand fractions and a decrease in the clay fraction were observed in the sediments deposited within the periods of the 1080s–1120s, the 1300s–1310s, 1400s, 1460s and the 1690s–1700s. The sediments that accumulated in the 1790s were mainly composed of sand, fine silt and coarse silt, with average compositions of 43.2%, 23.7% and 21.3%, respectively. After the 1800s, the coarse silt and sand fractions gradually decreased but remained relatively high.

The magnetic susceptibility (MS) values range from 28.6 to 70.4 10^−8^ m^3^ kg^−1^, with an average of 33.7 10^−8^ m^3^ kg^−1^ (Fig. 3). The MS was low in the sediments that accumulated within the period of the 1080s–1790s, although higher MS values were recorded in the 1080s–1100s, 1160s, 1300s, 1400s, 1460s, 1560s, and 1700s. The maximum MS occurred in the 1700s and showed dramatic fluctuations from the 1700s to 2012 AD.

### Climatic implications of proxies in Basomtso Lake

The LOI_550_ is an effective proxy for estimating the organic content of lake sediments^22^ and thus has been widely used to reconstruct past climatic and environmental conditions^23^,^24^. The C/N ratio of lacustrine organic matter can be used to distinguish autochthonous and allochthonous sources^24^. The C/N ratios in BSCW-1 ranged from 15 to 25, indicating that the organic material was mainly derived from terrestrial plants^24^ (Fig. 3). Recent studies of lakes located on the southeastern TP have suggested that the organic matter content in the sediment is related to the mean annual precipitation^25^. However, present-day glaciers are distributed throughout the Basomtso Lake basin. These glaciers can affect the regional precipitation, with increased precipitation above the snow line and reduced precipitation in the adjacent valley areas^26^. The increase of glacier melt water caused by a warmer climate can transport more terrestrial plant debris and nutrient materials into the lake^25^, resulting in a higher organic content, as indicated by higher LOI, TN andTOC values (Fig. 3).

In glacier-fed lakes, the sedimentary supply not only depends on the size and activity of glaciers, but also the transport capacity of the glacial melt water. Fluctuations of the glaciers on the southeastern TP are mainly controlled by temperature changes^27^,^28^. Although several glacier studies have reported an increased erosion rate with glacier advance, glacier retreat may also cause higher sediment variability and erosion through the exposure of sub-glacial sediment^29^,^30^,^31^. An increased transport capacity related to increased fluvial discharge in the warm season has been reported in glacial and snow-covered basins^31^,^32^. The southeastern TP is mainly affected by the ISM, which is assumed to be driven by summer ocean-continent temperature gradients. In the warm periods, the increased fluvial runoff related to increased glacier melt water^31^,^33^, as well as the intensified monsoon precipitation, results in a high-energy transport and deposition environment^31^. Thus, more clastics can be transported into the lower reaches of Basomtso Lake, resulting in high contents of coarse silt and sand as well as high MS values in the sediment. Therefore, we suggest that the higher organic matter content, sand and coarse silt fractions, and MS during the past millennium likely reflects a higher sediment input, warm conditions and higher glacier melt water input in the Basomtso Lake region, and vice versa.

### Climate changes over the past millennium in the Basomtso Lake region

The climate variations since the 1080s on the southeastern TP were reconstructed based on the AMS ^14^C dating and the analysis of LOI, TOC, TN, grain size and MS in the sediment of Basomtso Lake (Fig. 3). The high values of LOI, TOC, TN, coarse silt, sand and MS in the sediment indicated higher sediment input, likely warmer climate conditions and higher glacier melt water input during the 1080s–1140s and during the 1790s–2012 AD, which corresponded well with the MCA and CWP, respectively. By contrast, a lower sediment input and cold climate were likely prevalent during the 1140s–1790s, which correlated well with the LIA. The duration of the LIA was consistent with previous studies suggesting that the alpine glaciers in the vicinity of the eastern Nyainqentanglha Range generally advanced^27^,^28^,^34^,^35^. By the mid-18th century, the glaciers in the eastern Nyainqentanglha Range reached the maximum extent of the LIA glaciers^27^, after which a warming trend was recorded. Tree ring and ice core data for the central and southern TP indicate a warm and dry climate around the 1790s^36^,^37^,^38^ in which the glacier melt intensified, resulting in an increase of the areas of pre-glacial lakes on the central and southern TP^20^,^39^. The rapid melting of glaciers in around the 1790s should have resulted in enhanced fluvial runoff, transporting the sand fraction and forming the sand sediment layer in Basomtso Lake. The well-sorted fine sand in the sand layer closely resembles the sediment from dead-ice bodies^27^, indicating a rapid glacial retreat. We therefore suggest that the rapid warming around the 1790s indicates the end of the LIA on the southeastern TP.

Evidence of frequent climate fluctuations over the past millennium at the decadal time scale was well documented in our record (Fig. 3). Five distinct episodes with higher sediment input, likely warmer conditions and increased glacier melt water input, characterized by high organic matter, coarse silt, sand and MS levels, were recorded in the 1080s–1100s (I), 1390s–1400s (II), 1450s–1460s (III), 1700s–1720s (IV), and 1800s–1870s (V). Seven episodes with lower sediment input and likely colder conditions, indicated by low organic matter levels, coarse silt fractions and MS values were recorded in the 1150s–1160s (1), 1260s–1270s (2), 1340s–1360s (3), 1420s–1440s (4), 1560s–1580s (5), 1660s–1670s (6), and 1740s–1780s (7). The episodes with lower sediment input were closely followed by the periods with higher sediment input and likely warmer conditions (Fig. 3), which were then followed by periods of gradual cooling and lower sediment input. Both the episodes with higher and lower sediment input are characterized by rapid changes and short time durations. Previous studies suggested the occurrence of regional glacier advances in the eastern Nyainqentanglha Range, and hence, presumably, cooler temperature around the 1400s–1430s, 1500s–1540s, 1580s–1590s, 1650s–1680s, 1740s, and 1800s^27^,^28^,^34^,^35^. The episodes with lower sediment input and likely colder conditions in the Basomtso Lake record were generally consistent with the regional glacier advances. The episodes with higher sediment input associated with the II, III, and IV time periods, as well as the seven episodes with lower sediment input, generally occurred within the LIA, indicating that the climate was variable under cold climate conditions.

Nonsynchronous increases in silt and coarse silt fractions, MS values and organic matters in the sediment deposited around the 1160s and the 1300s have been recorded in the Basomtso Lake. This would argue that not all the coarser-grained layers reflect the same changes in the glacier dynamics or the watershed, may be with the occurrence of different thresholds of glacial melt-water/sediment reaching the lake. Therefore, we did not interpret these two episodes in Basomtso Lake record. Decreasing LOI, coarse silt, sand and MS levels have been recorded in recent decades, superficially indicating a lower sediment input and a likely trend of cooler conditions, which is inconsistent with the increasing temperature on the southeastern TP^16^,^38^. This contradiction should correspond to glacier retreat and anthropogenic activities. The glaciers on the southeastern TP have retreated substantially over the past 30 years^40^. The shrinkage of glaciers and the increased sediment delivery distances might cause the LOI, grain size and MS in the distal glacier lakes to be less sensitive to glacial signals^30^,^33^. Moreover, the “Chongjiu Dam” was built in Basomtso Lake in 2002 AD, which raised the lake level by 2 meters. The higher lake level together with water control by the “Chongjiu Dam” should have resulted in a more subdued transport and deposition environment in Basomtso Lake.

### Potential driving factors

The LOI is significantly correlated with the TOC (r = 0.69, p < 0.001, n = 118), TN (r = 0.78, p < 0.001, n = 118), coarse silt (r = 0.25, p < 0.001, and n = 479) and MS levels (r = 0.11, p < 0.015, n = 479). We therefore used the LOI to perform spectral analysis. The result indicates a significant low frequency variability of 113–120 years (>95% confidence level) (Fig. 4), which is similar to that detected in climate reconstructions from the TP^13^,^37^ and the Arabian Sea^41^. A cycle of 113–120 years has been reported in total solar irradiation (TSI) data derived from sunspot numbers^42^, as well as ^10^Be and ^14^C data^43^, and may be correlated with a Gleissberg cycle of solar activity^13^,^44^. Variations of solar irradiation have a considerable influence on the climate over the continents in the Northern Hemisphere^12^,^42^,^45^. Therefore, the climate variations in Basomtso Lake may be related to changes in solar activity during the past millennium.

## Discussion

In view of long-term climate evolution, climate fluctuations exist at different time scales (e.g., glacial–interglacial cycles^46^, ice raft events in the North Atlantic^4^, the Atlantic Multi-decadal Oscillation^47^, and the Pacific Decadal Oscillation^48^). Similarly, the climate on the TP was variable over the past millennium^5^,^11^,^19^. High-resolution tree ring and ice core data have indicated generally colder or warmer episodes in different study areas of the TP during the past millennium^5^,^16^,^38^,^49^,^50^. However, the climate fluctuations inferred from different reconstructions (including the Basomtso Lake record) are occasionally inconsistent with respect to durations and volatilities. Three reasons may contribute to this lack of consistency: differences in the regional climate related to the varied geographical patterns; proxies that vary in their climatic sensitivity; and dating uncertainties.

The organic matter content, grain size and MS in Basomtso Lake are sensitive to glacier activities that are directly forced by climate variations. In the eastern Nyainqentanglha Rage, a comparison between the glacier fluctuations and the regional reconstructions of temperature and precipitation suggested that temperature likely played a dominant role in forcing glacier advances in last millennium, whereas precipitation changes probably played a subordinate role^27^. We have considered the possibility that the higher sediment input may be related to flood events associated with heavily precipitation; however, there are several factors that strongly dispute this explanation. First, the increased levels of LOI, coarse silt, sand and MS in the coarser-grained layers are different than that of the flood layers, in which rapid sediment deposition should have diluted the organic matter^51^. Second, clay caps cannot be found in the coarser-grained layers. If the coarser-grained layers were related to flood events, then clay caps should be found in the upper part of the flood layer as the flood event diminished^51^. After the 1830s, the LOI, coarse silt, sand and MS contents remained high in the sediments, which also contradicts the occurrence of flood layer deposition. We therefore suggest that the higher sediment input in Basomtso Lake is likely related primarily to increased glacier melt water under warmer conditions.

During the LIA, there were lower levels of organic matter, coarse silt and sand fractions, and MS but with a higher clay fraction, which is a reflection of reduced sediment input that likely occurred in a cold climate and with lower glacier melt water supply. This assumption may be challenged by LIA glacier advances. As the glacier grew, the increased water discharge and the shortened delivery distance made it easier to transport clastics into the distal glacier lakes^29^,^52^. Three main phases of LIA glacier advances in the eastern Nyainqentanglha Range have been deduced: an early phase before 1500 AD, an intermediate phase from 1650 to 1740 AD consisting of several strong advances and intermediary slightly retreats, and a late phase manifested in a set of inner recessional moraines after 1800 AD^27^. The glacier advances from the mid-17th century to the mid-18th century represented the maximum glacier extent in eastern Nyainqentanglha Range during the Holocene^27^,^35^. The extended glaciers should have intensified glacier abrasion and shortened the transport distance to Basomtso Lake. However, variations in grain size, MS and LOI in the sediment of Basomtso Lake suggest that the influence of the glacier advances during the LIA should have been limited, probably due to insufficient glacier melt water or the limited dimensions of the glacier advances. Three episodes with higher sediment input and a sandy layer have been recorded during and at the end of the LIA, suggesting that the LOI, coarse silt and sand fractions as well as the MS should have been more sensitive to the glacier retreat and intensified glacier melt water.

Earthquakes may also have an influence on sedimentary process because the sub-aqueous slope failures (as well as basin slope failures) and reworking of sediments during a strong earthquake^53^,^54^ can substantially affect the sedimentary texture, forming homogenite with distinct grain size compositions and MS values^54^,^55^. According to the China Seismic Information (http://www.csi.ac.cn), the paleo-seismic events in Basomtso Lake basin were limited after 1050 AD. However, a 7.0 magnitude earthquake in 1642 AD (95°36′ E, 30°48′ N, approximately 180 km east of Basomtso Lake) and an 8.6 magnitude earthquake in Assam in 1950 AD (96°42′ E, 28°24′ N, approximately 320 km southeast of Basomtso Lake) were recorded (Fig. 3). By contrast, no evidence of earthquakes was correlated with proxy variations in Basomtso Lake around the 1640s or 1950s (Fig. 3). Therefore, the rapid variations in organic matter content, grain size and MS in the core sediment of Basomtso Lake are primarily controlled by climate changes but not paleo-seismic events.

The Basomtso Lake record is significantly correlated with the Nyingchi mean January–June temperature anomaly inferred from tree ring data from the southeastern TP^16^ (Fig. 5c), suggesting that the sediment input in Basomtso Lake is related to the mean January-June temperature on the southeastern TP. The episodes with higher/lower sediment input in the Basomtso Lake record are also consistent with other climate reconstructions^49^,^56^. Our reconstruction is also correlated with temperature changes in the Northern Hemisphere^7^ (Fig. 5d), which suggests that the variations in sediment input of the Basomtso Lake record are sensitive to global temperature variations. However, the most striking feature of our record is the detailed climate fluctuations characterized by abrupt changes and short time durations. Rapid climate fluctuations over the past several hundred years have been reported for the TP^5^,^38^,^49^,^50^, suggesting that the TP is sensitive to climate fluctuations at decadal to annual time scales. Further research is needed to confirm these rapid climate fluctuations on the TP as well as worldwide.

## Methods

**Figure 1:**
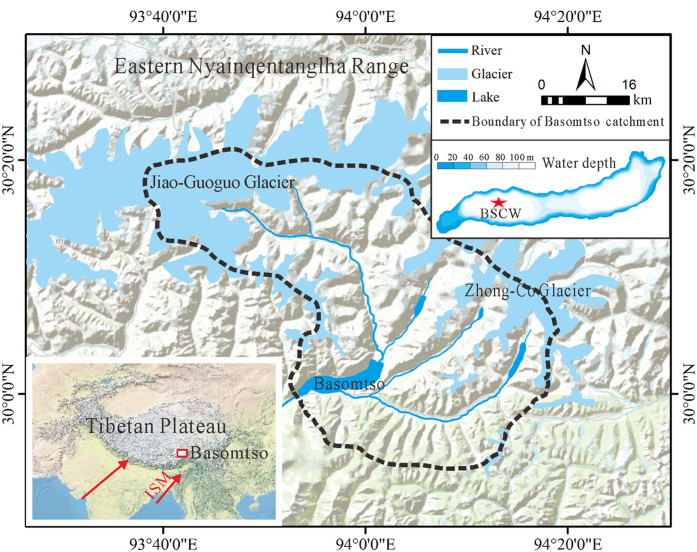
Map of the location of the Basomtso Lake basin (including the watershed, locations of glaciers and drainage network, the core site and a bathymetric map of the lake). The inset shows the main atmospheric circulation systems influencing the Basomtso Lake basin. The terrain map was generated using ArcMap 10 software (ESRI, USA, http://www.esri.com,) based on the basemap of USA Topo Maps (http://goto.arcgisonline.com/maps/USA_Topo_Maps). The bathymetry was measured in 2012 AD and bathymetry contours were plotted using Surfer 9.0 software (http://www.goldensoftware.com).

**Figure 2:**
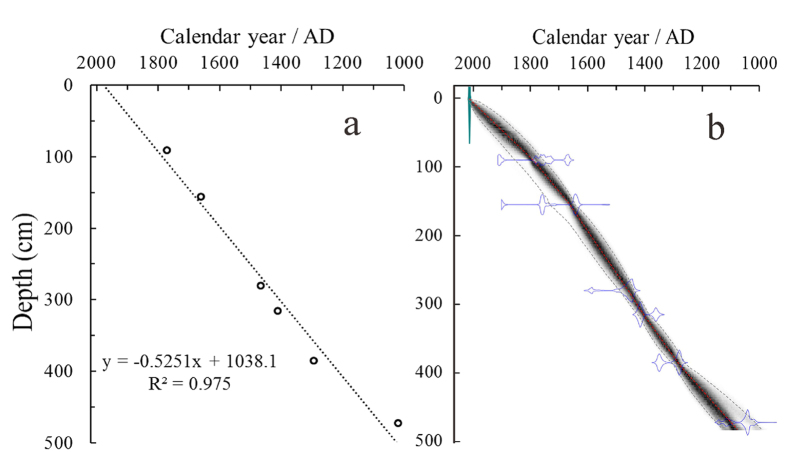
(**a**) The six AMS ^14^C dataset and their distribution with depth. (**b**) Age-depth model of BSCW-1, black dots indicate the 95% probability intervals of the model.

**Figure 3:**
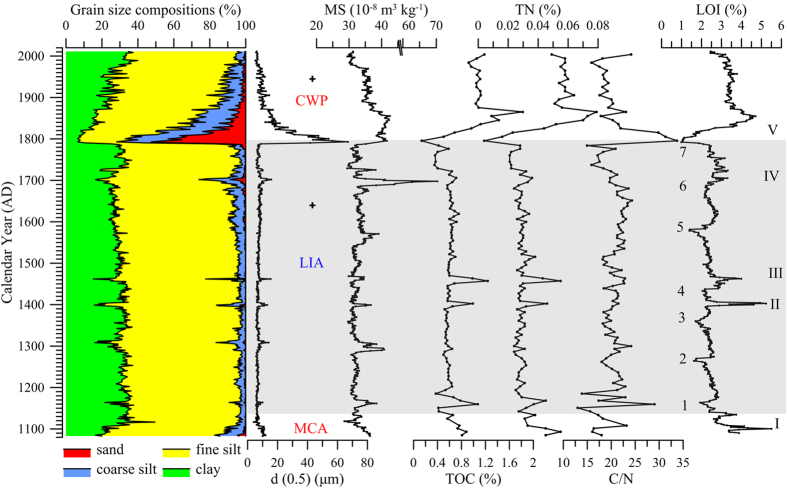
Variations of grain size compositions, median size d (0.5), MS, TOC, TN, C/N and LOI in the core sediment of Basomtso Lake. I, II, III, IV and V indicate episodes with higher sediment input and likely warmer conditions characterized by higher LOI, TN, TOC, silt and sand fractions and MS values, and 1, 2, 3, 4, 5, 6, and 7 indicate episodes with lower sediment input and likely colder conditions characterized by lower LOI, TN, TOC, silt and sand fractions and MS values. Two cross-shaped marks indicate strong earthquakes recorded in the southeastern TP.

**Figure 4:**
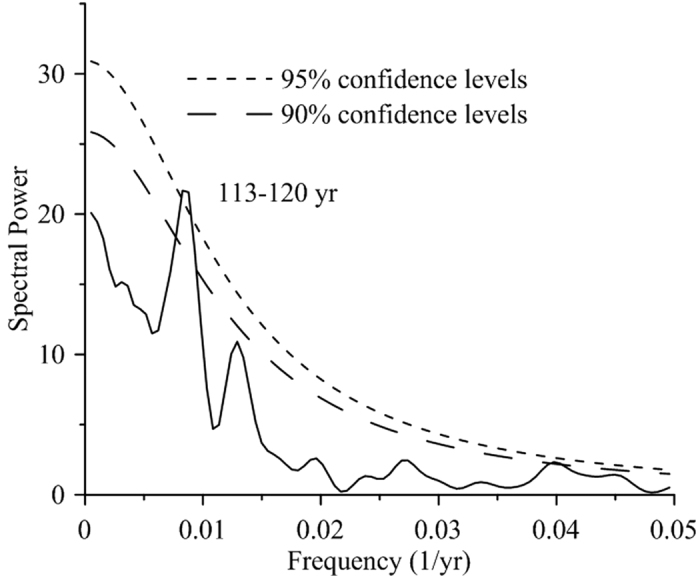
Spectral Analysis results for the LOI record from Basomtso Lake over the past millennium. Peaks are labelled with their periods in years above 95% confidence level.

**Figure 5:**
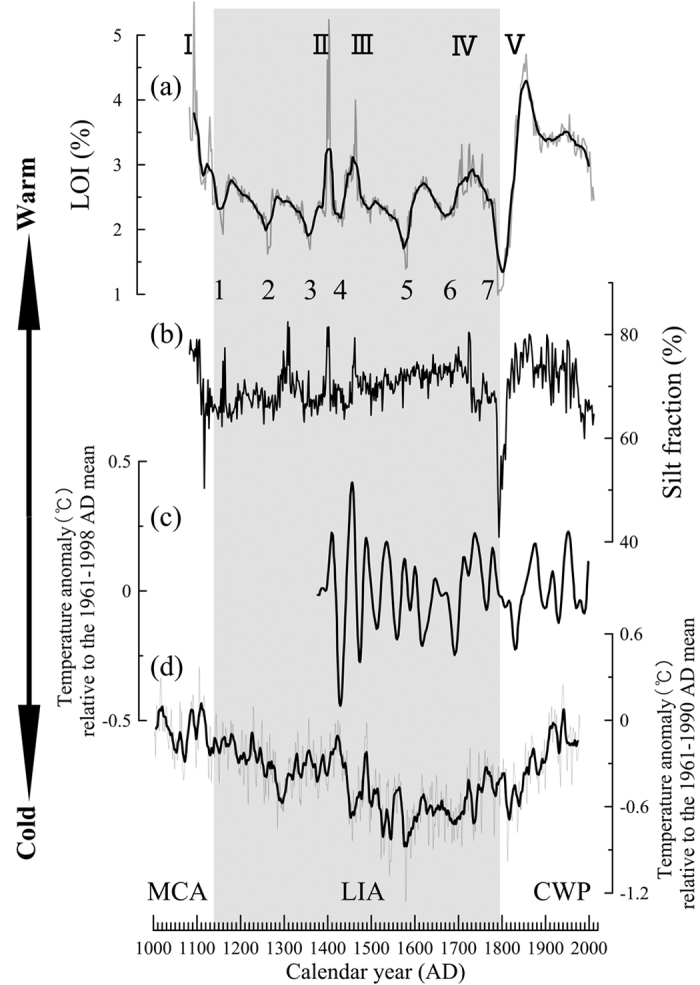
Climate fluctuations in Basomtso Lake recorded by (**a**) LOI and (**b**) silt fractions and their comparisons with (**c**) the temperature anomaly from Nyingchi^16^ and (**d**) the temperature anomaly from the Northern Hemisphere^7^. Black lines in (**a,b,d**) are 11-yr running averages.

In October 2012, two parallel sediment cores (BSCW-1 and BSCW-2) were retrieved from Basomtso Lake at a water depth of 120 m (Fig. 1, 93°55′44.4″ E, 30°1′4.8″ N) using a UWITEC sample system. The sediment cores were transported to the State Key Laboratory of Lake Science and Environment, Nanjing Institute of Geography and Limnology, Chinese Academy of Sciences and kept at 3.9 °C until analysis. The longer sediment core, BSCW-1 (4.82 m), was split, photographed and visually described in the laboratory. BSCW-1 primarily consisted of medium- to fine-graded laminates composed of grey fine silt and clay, whereas increased contents of coarse silt and sand were observed in the sections of the core at depth of 1.35–1.40 m, 2.78–2.82 m, 3.18–3.22 m, 3.71–3.76 m, and 4.71–4.82 m. The 0.8–0.9 m section was a well-sorted sand layer. This layer was clearly distinct compared with the sediment in the lower sections but could not be distinguished from the overlying sediment. The samples were separated at 1-cm intervals, freeze-dried, and used for LOI, TOC, TN, grain size, and MS analyses.

A few macrofossils (typically twigs and intact leaves) were found in the coarser-grained sediment layers. Six well-preserved leaves identified at different depths in BSCW-1 were removed for AMS ^14^C measurements at the Rafter Radiocarbon Laboratory, Institute of Geological and Nuclear Sciences (GNS), New Zealand. Calendar year ages were determined by calibration of radiocarbon dates using CALIB 6.0^57^ with an IntCal13 calibration dataset^58^.

Samples for LOI analysis were dried overnight at 105 °C, cooled and weighed (W_105_). They were then ignited at 550 °C for 4 h, cooled and weighed (W_550_)^22^. The samples were cooled in a desiccator. The LOI_550_ (weight percentage) was calculated as follows^22^:


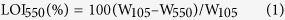


The sediments (collected at 4-cm intervals) were pre-treated with 10% HCl to remove carbonates and then used to measure the TOC and TN contents using an EA3000 Elemental Analyzer. Replicate analyses of well-mixed samples showed that the precision was ca. ± <0.1% (1 standard deviation (SD)). The C/N ratios were derived from these data.

For grain size determination, the samples were pre-treated with 10% H_2_O_2_ and then 10% HCl to remove organism and carbonate, and each of these steps was followed by a standing period of over 24 hours. Approximately 10 ml of 10% Na_2_P_2_O_7_ was then added to the remaining sample, which was then kept in sonic oscillation for 15 minutes prior to the grain size measurements. The grain size composition was determined using a Malvern Instruments Mastersizer-2000 laser diffraction particle size analyser (Malvern Instruments Ltd., UK).

The freeze-dried samples were powdered and packed into 8-cm^3^ plastic boxes, and the initial net weights were then measured. The MS was measured using a Bartington MS-2 susceptibility meter. The measurements were repeated 4 times, and the average value in volume-specific SI units was normalized to sample mass.

The correlation analysis function in SPSS 19.0 software for Windows (SPSS Inc., USA) was used to analyze the relationships between the proxies. Spectral analysis was applied using REDFIT 3.8 to examine the characteristics of the climate variability in the frequency domain.

## Additional Information

**How to cite this article**: Li, K. *et al.* Rapid climate fluctuations over the past millennium: evidence from a lacustrine record of Basomtso Lake, southeastern Tibetan Plateau. *Sci. Rep.* **6**, 24806; doi: 10.1038/srep24806 (2016).

## Figures and Tables

**Table 1:** AMS ^14^C dates of BSCW-1 together with the sampling depth and dating materials.

Sample ID	Depth (cm)	Material	Lab. No. NZA-	δ^13^C%	AMS ^14^C Age(yr BP ± 1σ)	Calibrated age[yr BP with 2σ]	Median ageCalendar yr
BSCW1-1-91	90–91	Leaves	57120	−27.3 ± 0.2	184 ± 18	144–216	1770
BSCW1-3-50	155–156	Leaves	57643	−27.0 ± 0.2	228 ± 20	277–305	1659
BSCW1-3-175	280−281	Leaves	57644	−27.6 ± 0.2	410 ± 21	456–512	1466
BSCW1-4-34	315–316	Leaves	57645	−26.3 ± 0.2	545 ± 21	521–559	1410
BSCW1-4-104	385–386	Leaves	58786	−24.9 ± 0.2	675 ± 22	640–674	1293
BSCW1-4-191	472–473	Leaves	57646	−26.6 ± 0.2	993 ± 21	903–957	1020
